# Multivariate Granger causality unveils directed parietal to prefrontal cortex connectivity during task-free MRI

**DOI:** 10.1038/s41598-018-23996-x

**Published:** 2018-04-03

**Authors:** Andrea Duggento, Luca Passamonti, Gaetano Valenza, Riccardo Barbieri, Maria Guerrisi, Nicola Toschi

**Affiliations:** 1 Department of Biomedicine and Prevention, University of Rome Tor Vergata, Rome, Italy; 2 Institute of Bioimaging and Molecular Physiology, National Research Council, Catanzaro, Italy; 3 Department of Clinical Neurosciences, University of Cambridge, Cambridge, UK; 4 Bioengineering and Robotics Research Centre “E. Piaggio”, and the Department of Information Engineering, University of Pisa, Pisa, Italy; 5 Department of Electronics, Informatics and Bioengineering, Politecnico di Milano, Milano, Italy; 6 Department of Anesthesia, Massachusetts General Hospital, Boston, Massachusetts USA; 7 Department of Radiology, Athinoula A. Martinos Center for Biomedical Imaging, Massachusetts General Hospital and Harvard Medical School, Boston, Massachusetts USA

## Abstract

While a large body of research has focused on the study of functional brain “connectivity”, few investigators have focused on directionality of brain-brain interactions which, in spite of the mostly bidirectional anatomical substrates, cannot be assumed to be symmetrical. We employ a multivariate Granger Causality-based approach to estimating directed in-network interactions and quantify its advantages using extensive realistic synthetic BOLD data simulations to match Human Connectome Project (HCP) data specification. We then apply our framework to resting state functional MRI (rs-fMRI) data provided by the HCP to estimate the directed connectome of the human brain. We show that the functional interactions between parietal and prefrontal cortices commonly observed in rs-fMRI studies are not symmetrical, but consists of directional connectivity from parietal areas to prefrontal cortices rather than *vice versa*. These effects are localized within the same hemisphere and do not generalize to cross-hemispheric functional interactions. Our data are consistent with neurophysiological evidence that posterior parietal cortices involved in processing and integration of multi-sensory information modulate the function of more anterior prefrontal regions implicated in action control and goal-directed behaviour. The directionality of functional connectivity can provide an additional layer of information in interpreting rs-fMRI studies both in health and disease.

## Introduction

In recent years, the notable progress in functional MRI (fMRI) technology and the advent of high-field (3T) and ultra-high (7T) field neuroimaging has enormously fuelled the study of the *functional connectome* (i.e. the dependencies between statistical fluctuations in localized, regional brain activity) and its disease-related abnormalities. More specifically, functional brain connectivity measures refer to statistical relationships between non-spatially-contiguous neurophysiological events, and this type of spatio-temporal synchronization patterns has been widely established as a proxy for characterizing the organization of higher-order brain function in the human brain. In this context, coordinated research efforts like the Human Connectome Project (HCP) are pushing the boundaries of data collection methodologies as well as cohort sizes, hence providing access to unprecedented spatiotemporal resolution as well as cohort sizes in extremely well-characterized samples of healthy individuals.

While most fMRI connectivity studies are still based on linear or nonlinear measures of undirected correlation, estimating the directionality of whole-brain connectivity is an important and largely unaddressed issue. In detail, while underlying anatomical pathways are bi-directional in most circumstances (especially in cortico-cortical connections), it cannot be assumed a priori that commonly observed resting state fMRI (rs-fMRI) connectivity patterns between brain networks (e.g., between parietal and prefrontal circuits) are necessarily symmetrical (e.g., that connectivity effects from parietal areas to prefrontal cortices are comparable to effects in the reverse direction). To enhance our knowledge of functional brain organization as well as possible hierarchical connectivity patterns, Multivariate Granger Causality (MVGC) approaches have recently been employed to incorporate information about the directionality of the influence exerted by a brain region (or circuit) on another. In brief, a dynamical system is said to Granger-cause another if information from the past of the former allows better prediction of the future of the latter, compared to predictions based on the past of the latter alone^1^. This general definition allowed the study of time-domain causality^2^ and the definition of a measure of directed feedback between time-series^3^. As a result of these seminal studies, the idea of GC has been commonly associated with its implementation in terms of autoregressive (AR) models^4^ in the univariate case and of vector AR or MVAR models^5^ in the multivariate case. This also includes the possibility to account for zero-lag causality (i.e. the instantaneous influence of one variable on another)^6–8^.

In the context of fMRI, the early studies by Goebels *et al*.^9^ and Harrison *et al*.^10^ pioneered the use of MVAR models within a framework which is now commonly employed in fMRI analysis^11,12^ (see ref.^13^ for review). Compared to electroencephalography (EEG) data (which has been traditionally employed for connectivity estimation), one of the main advantages of fMRI is the spatial specificity in quantifying functional, time-resolved brain activity with a resolution of about 2 mm^3^ (which in turn results in approximately 10^5^ time-resolved signals for every fMRI dataset). This comes at the cost of temporal resolution, and indeed fMRI signals are commonly acquired at around 1 Hz. Due to constraints on in-scanner subject time, fMRI data also suffers from short data lengths (of the order of minutes or tens of minutes), and the use of fast Echo-Planar Imaging (EPI) techniques commonly results low signal-to-noise ratio (SNR)^14^.

The multivariate nature of brain signals recently lead to the employment of partial correlation approaches when studying fMRI connectivity^15,16^ and highlights the importance “conditioning” GC estimates- i.e., the idea of estimating their “true” directed interaction between two brain regions net of all other, indirect modulation pathways^17^. To this end, partial conditioning based on *a priori* identification of the subset of signals which share the most information with the signal under study has been applied to the study of brain connectivity^18^. While this *prior* down-selection can provide a practical strategy to alleviate the issue of limited lengths of typical fMRI datasets, given the high density and complexity of brain networks^19,20^, in order to correctly eliminate spurious connections GC analyses of fMRI signals should include the widest possible set of conditioning variables, i.e., be performed on the whole brain^5,11–13,21^. Nonlinear methods have also been introduced for a better estimation of causality^22,23^. The analysis of causality of the neuronal activity by use of the intrinsically low temporal resolution BOLD data is challenging also considering that, due to the very nature of the BOLD acquisition, neuronal activity is convolved with a slow-varying haemodynamic response function (HRF) which further provide a temporal confound. The use of Multivariate Granger Causality (MVGC) in estimating directed BOLD connectivity is therefore controversial as it has been seen to be somewhat fragile to undersampling^21^, albeit it is robust to haemodynamic convolution.

The aim of this study was: (a) to using extensive synthetic data simulations and MVGC estimates^11,21,24–28^ of signals generated from complex networks to quantify its suitability for applications to BOLD data (b) to apply the conditioned causality framework to data made available by the HCP consortium (whose volume and quality allows to alleviate some of the typical concerns related to the use of GC methods in fMRI) in order to estimate and characterize top 1% the directed functional connectome of the human brain.

## Results

### Synthetic networks

**Figure 1:**
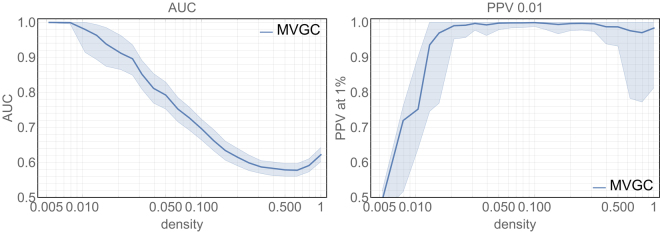
Left: Area under the ROC curve (AUC) obtained when employing MVGC. For any given network density the full line and the shadowed area represent the median and the interquartile ranges ad extremes (respectively) across 10 realizations of a pool of 32 different networks (see Methods). Right: PPV when the strongest 1% detected link are considered. For any given network density the full line and the shawdowed area represent the median and the interquartile ranges ad extremes (respectively) across 10 realizations of a pool of 32 different networks (see Methods).

Figure 1 summarises the main results of MVGC in synthetic networks in terms of detection AUC and top 1% PPV performance. For any given network density, the median and the quartiles across all 10 realizations of all 32 networks are depicted (i.e. 320 realizations per point) for AUC and the PPV of the strongest 1% estimated links (akin to the strategy we adopt in real data in the second part of the paper). Most notably, the AUC for the MVGC-based detection is extremely high (AUC > 0.9) for every density below than 0.2, and decrease steeply and almost monotonically with increasing network density, while the PPV has a sharp rise at low densities and keeps near unity (i.e. almost no false positives) for all densities greater than 0.02. Only for densities in the interval [0.015; 0.02] both AUC and PPV at 1% are larger than 0.9.

### *In-vivo* causal connectome of the human brain

**Figure 2:**
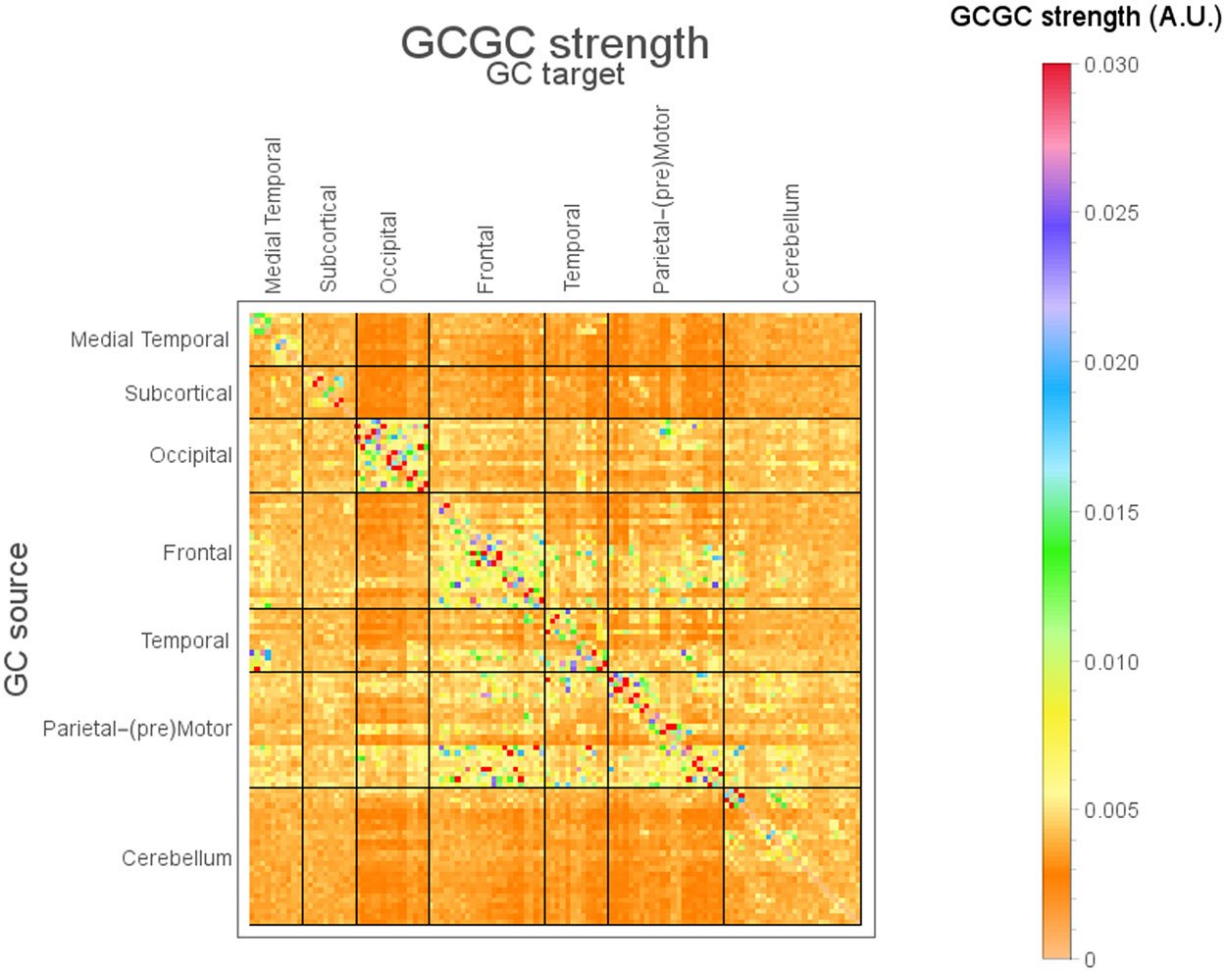
Full MVGC between all 116 AAL ROIs, computed as the overall median for 100 unrelated subjects (4 sessions per subject of 1200 volumes each). “GC source”: driving signal, “GC target”: driven (i.e. caused) signal.

**Figure 3:**
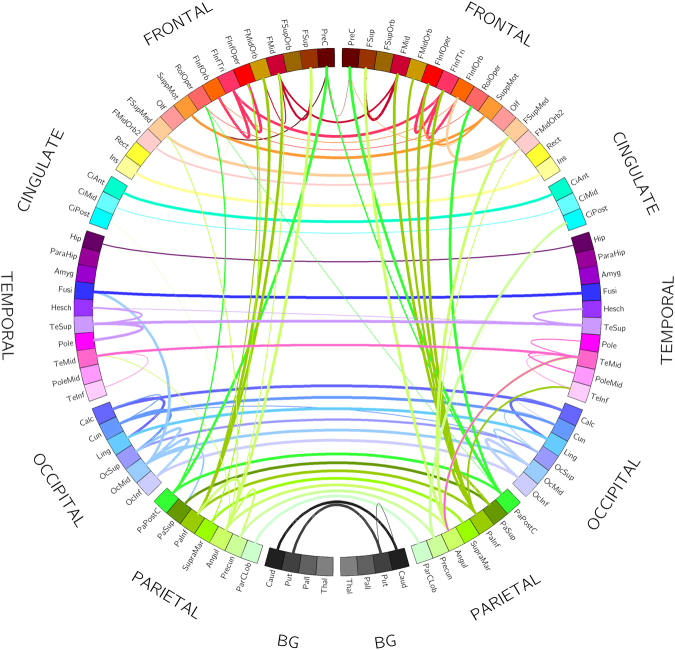
MVGC between all 116 AAL ROIs, computed as the overall median for 100 unrelated subjects (4 sessions per subject of 1200 volumes each) and thresholded at the top 1% percentile in strength. Every edge (i.e. connection) is coloured according to the node (i.e. ROI) which is causing the other node. The width of each edge if proportional to the strength of the connection.

**Figure 4:**
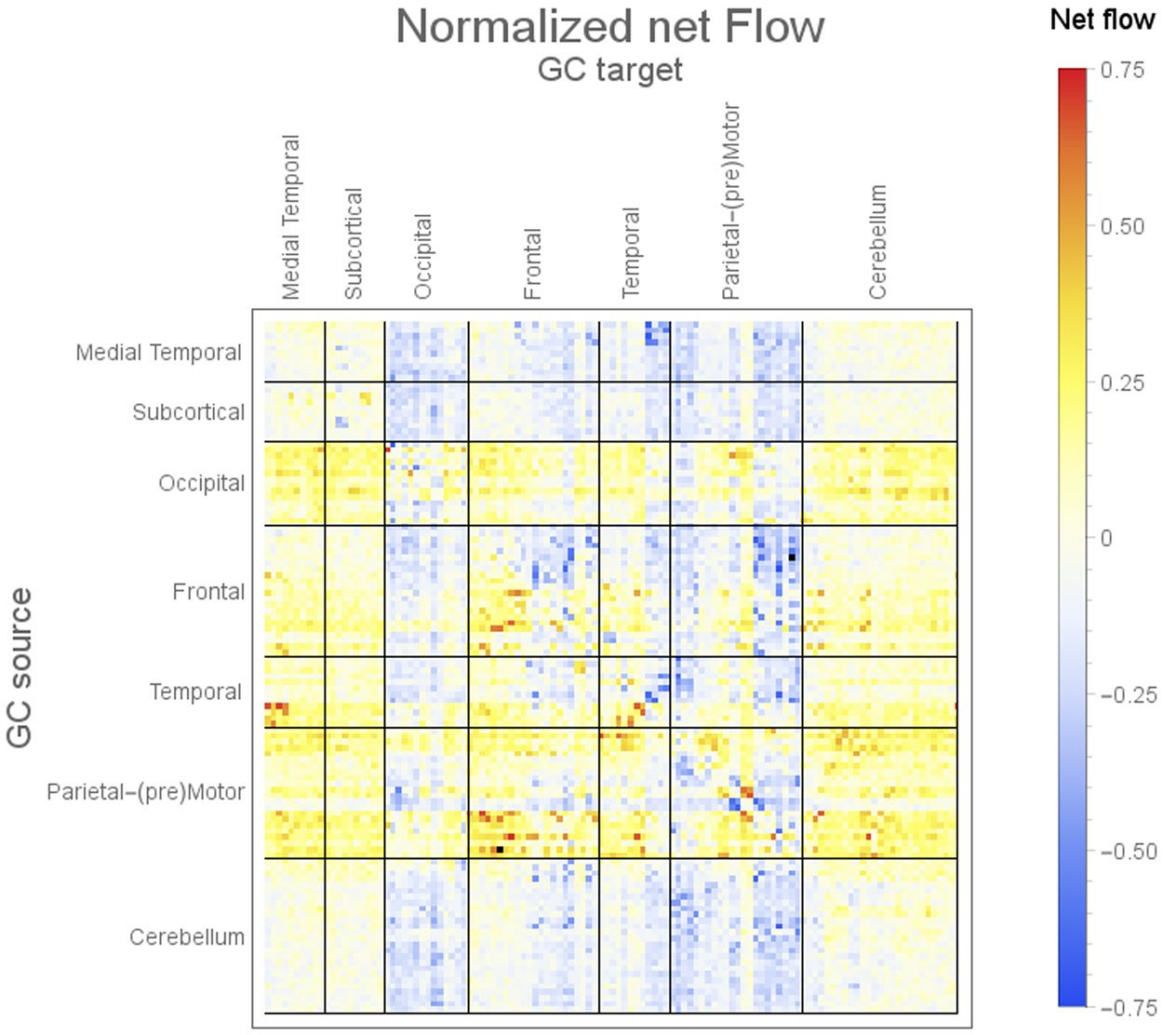
Full normalized flow ([G − G^T^]/[G + G^T^]) (unitless, G is the matrix depicted in Fig. 2) between all 116 AAL ROIs, computed as the overall median for 100 unrelated subjects (4 sessions per subject of 1200 volumes each). “GC source”: driving signal, “GC target”: driven (i.e. caused) signal.

**Figure 5:**
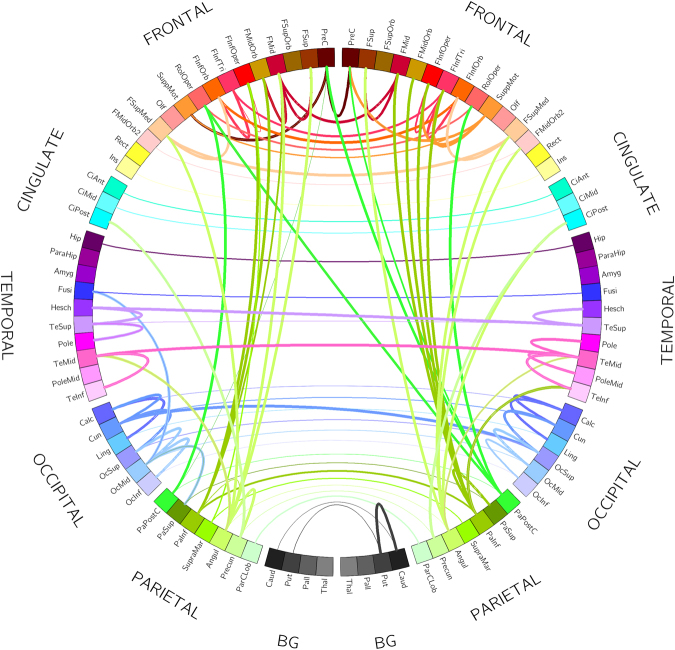
Normalized information flow F = ([G − G^T^]/[G + G^T^]) for connections shown in Fig. 4. Only the top 1% of the strongest MVGC connections are displayed. Every edge (i.e. connection) is coloured according to the node (i.e. ROI) which is causing the other node. The width of each edge if proportional to the flow F.

Figures 2 and 3 summarize the main findings of MVGC analysis in HCP data from 100 healthy subjects. Figure 2 shows the elements of the inter-subject median MVGC matrix between the 116 ROIs extracted from the AAL atlas, and Fig. 3 shows the top 1% connections (in terms of MVGC strength which, according to our synthetic simulations, has a PPV of approximately 1) of this matrix in a circular plot where the ROIs are displayed in an anatomically meaningful order. Figure 4 show the inter-subject median of normalized causality flow ***F****,* and Fig. 5 shows the flow ***F*** for the top 1% connections shown in Fig. 4.

Overall, intra-hemisphere causality flow dominates with respect to inter-hemisphere causality flow. Causal, intra-hemispheric parietal-to-frontal and parietal-to-cingulate connections score among the most non-symmetric (i.e. strongly directional) connections. Also, a strong intra-hemisphere causal flow is noticeable from the inferior parietal lobule to the lateral part of the middle frontal gyrus (both left and right), the orbital middle frontal gyrus (both left and right), and opercular part of inferior frontal gyrus (both left and right). Also, a similarly strong causality flow, however confined to the right hemisphere, is visible from the lateral postcentral gyrus (parietal cortex) to the Rolandic Operculum (frontal cortex) and its contralateral (left) precentral gyrus (frontal cortex). In the parietal cortex, parietal angular gyri (both left and right) are involved in a strongly asymmetric causal relationship towards the medial orbital superior frontal gyrus and to the posterior part of the cingulate gyrus. Few, and generally less strong, inter-hemisphere causality flow links were detected. Most notably: parietal-to-parietal links (from right middle temporal gyrus to the left middle temporal gyrus of the temporal pole; from superior temporal gyrus to its contralateral Heschl’s gyri); frontal-frontal and parietal to contralateral frontal connections were also detected (see Fig. 5 for details). We also found a noticeable amount of strong unidirectional causal connections among anatomically contiguous or semi-contiguous regions, e.g. middle temporal gyrus to its neighbouring temporal pole and inferior temporal gyrus (both left and right); and also from the inferior temporal gyrus to its neighbouring orbital middle frontal gyrus.

## Discussion

In this paper, we have employed extensive synthetic data simulation to explore the top 1% directed connections within complex, physiologically inspired directed neural networks of various densities, and successivley employed our detection method to explore the top 1% directed connectome using data from 100 unrelated subjects provided by the HCP consortium. In order to ensure the relevance of our simulations to the above goal, we employed massively parallel computational resources to substitute each network node with a tightly interlinked Izhikevich column/neural population, and have modelled each network link with realistic neuronal fiber bundles which exert their compounded influence on each other in a physiologically realistic and *a priori* identifiable directional way. The output of this neural model is then cascaded with a Baloon-model-like, structural model of neurovascular coupling. The compounded model is carefully parameterized and realized/evolved to reproduce our experimental conditions (number of data points = 1200, TR = 0.72s, etc) as closely as possible (see methods and supplementary information). This allowed to follow an approach similar to the one employed by Smith *et al*.^29^ while retaining full flexibility on network size and shape and simulation parameters.

We have confirmed that, while density is a dominant factor in determining the ability of reconstructing the ground truth network (with decreasing performance as density increases), the MVGC approach is able to deliver extremely high PPV (i.e. almost no false positives) if one is interested in the top 1% connections which, typically, already form an intricate network of their own. In this context, it should be noted that when reconstructing the functional whole-brain connectome based on fMRI data aggregated in a ROI-wise manner, one typically retains only 1–5% of connections for interpretation, which correspond to a limit when the small-world properties of a 90-nodes network, are still estimable^30^. Importantly, with few exceptions^31^, most previous papers which employ synthetic data simulations in the context of causality employ networks which are extremely small (3–7 nodes) and rather sparse with the number of edges *n*_*e*_ being of the same order of the number of nodes^32–34^. Other works that aim at large-scale Granger Causality on high dimensional networks employ usually ICA-based or PCA-based techniques to reduce the dimensionality^35,36^.

We subsequently applied our MVGC approach, including the realistically estimated autoregressive order 3, to human data, exploiting the unique time resolution as well as data volume afforded by the HCP dataset. In this context, while some authors^37,38^ discourage from the use of the use of time-domain filtering, it is unavoidable in order to remove gross confounds due to scanner heating and drift. It is important to note, however, that these data were denoised via a completely data-driven algorithm (ICA-FIX) which is based on an automated classifier specifically and manually trained to discern, amongst spatio-temporal independent component analysis results, diverse sources of noise (movement, physiological artefacts etc.). The version trained for HCP data has been seen to guarantee an accuracy (sensitivity and specificity) of around 99%^39,40^.

Importantly, the very nature of the BOLD signal, which stems from a convolution product between the neuronal activation and an unknown haemodynamic response function (HRF)^41^, poses some limits on the applicability of GC-based techniques in fMRI. However, the complex relationship between neuronal activation signal measured in fMRI is still not complete^42,43^. This relationship is thought to be highly variable between subjects^44^ as well as tissue-dependent both in humans^45^ and in animal models^46^, as well as possibly time-dependent^47^ and task-dependent^48^ (the largest source of fluctuations^49^ appears to be inter-subject variability). In this context, recent papers have used direct intracranial measurements of neural activity to explicitly investigate the effect on GC estimates of HRF-convolution-related confounds^50^, both using synthetic data simulations^21^ and in a subject population population^51^. These papers employed VAR models with articulate topologies as well as realistic haemodynamic models, and showed that GC is robust to HRF-related confounds. Instead, GC-based techniques in fMRI are more sensitive to signal down-sampling as well as signal quality (i.e. signal-to-noise ratio). Additionally, in the context of time-resolution^52^ employed both simulated (through the Balloon model)^53^ and real BOLD signals to demonstrate that combining time-domain and frequency domain information is able to alleviate the challenge of causality detection in the presence of significant HRF-related confounds. Still, we note that our model also exhibits nonlinear behaviour (like most physiological systems), and the reconstruction of the networks it generates could possibly benefit from the use of nonlinear methods^22,23^.

The MVGC whole-brain analyses in the large *in vivo* HCP data-set showed: (1) highly robust uni-directional functional connectivity interactions from *ipsilateral* parietal cortices to prefrontal regions (but *not viceversa*); (2) significant inter-hemispheric bi-directional functional connectivity between fronto-frontal, parieto-parietal, temporo-temporal (including medial temporal lobe regions), and occipito-occipital cortices; (3) significant *ipsilateral* functional interactions across areas belonging to the same frontal, parietal, temporal, and occipital circuits. Interestingly, the directionality of these functional patterns appears to follow either “bottom-up” patterns (i.e., from “hierarchically lower” to “hierarchically higher” cortices) or “top-down” pathways–i.e., from “hierarchically higher” to “hierarchically lower” cortices)^54^. For instance, we found evidence that the superior temporal gyrus (involved in early auditory processing along the Herschel gyrus) feed-forwarded to the temporal pole (implicated in more abstract representation of multisensory stimuli including linguistic and semantic representations)^55^. Likewise, activity in the calcarine cortex (which is a primary sensory area responding to relatively simple and “raw” visual information) was causally linked to activity in the lingual gyrus, which is in turn well-known to respond to progressively more complex visual inputs^54,56,57^. At the same time, our findings showed that the activity in “top-down” cognitive and “regulatory” regions including the superior frontal gyrus and middle frontal gyrus (which are often collectively referred as dorsolateral prefrontal cortex) were determining the rs-fMRI response in “limbic” PFC brain areas that have been consistently implicated in processing emotional stimuli as well as in coordinating autonomic responses and modulating affective behaviour (i.e., orbito frontal cortex and ventrolateral PFC areas)^54,58^.

Considered together, our results highlight that, in spite of the existence of mostly bi-directional anatomical pathways (especially in cortico-cortical connections), the directionality of the top 1% functional interactions commonly observed in rs-fMRI studies should not be necessarily assumed to be symmetrical^59^. In particular, while it is well established that the anatomical links between parietal cortices and prefrontal regions are strongly bi-directional in nature^60^, we found robust evidence of highly directional parietal-to-prefrontal causal connectivity effects rather than the opposite. Additionally, these uni-directional functional effects were localized within the same hemisphere and did not generalize or extend to cross-hemispheric functional interactions as it might have been assumed in earlier fMRI studies. Overall, these strong ipsi-lateral parieto-to-prefrontal functional interactions are consistent with a number of previous neuro-physiological studies showing that the activity within parietal cortices (a set of posterior regions involved in processing and integrating multi-sensory information) may have a driving role in determining the function of more anterior prefrontal areas, which in turn have been shown to be implicated in goal-directed behaviour as well as action control and planning^54,58^. On the other hand, the significant inter-hemispheric and bi-directional functional connectivity interactions between fronto-frontal, parieto-parietal, temporo-temporal (including medial temporal lobe), and occipito-occipital cortices are fully consistent with the strong inter-hemispheric anatomical links that exist between these areas, which are known to be primarily mediated by the corpus callosum^61^.

In addition, we found evidence of asymmetrical and ipsilateral functional interactions within brain regions belonging to the same lobe and possibly local micro-circuits (i.e., specific frontal-to-frontal, parietal-to-parietal, and temporal-to-temporal networks). Again, the directionality of some of these functional intra-lobar interactions appears to be organized in a “bottom-up” fashion, i.e. functional connectivity directed from “hierarchically lower” sensory cortices to “hierarchically higher” areas (see e.g., causal links from the calcarine cortex to the lingual gyrus). Nevertheless, our robust whole-brain MVGC analyses were also able to reveal more complex asymmetrical functional interactions between intra-lobular brain areas such as those identified from dorsolateral PFC regions to orbitofrontal areas and this is consistent with the regulating role of the former areas above the latter regions^58^. Last but not least, it should be noted that, although our rs-fMRI analyses did not involve psychometric or task-based measures and therefore could be overall considered as a “snapshot” of causal functional interactions in the whole-brain “at rest”, it is possible that some of the asymmetric functional patterns identified represent a fundamental brain mechanism that support key cognitive processes like multi-sensory integration or even high-level control of behavioural and emotional outputs^58^.

Some limitations of our work are worth mentioning. As noted by Ramsey *et al*.^62^, there are still a few open concern with the use of MVGC analysis in BOLD data. For example, while the search for alternative causality model can be astronomical and almost arbitrary, it is important to note that we achieve a PPV of almost 100% for the specific observables we are interested in. Likewise, while BOLD is an indirect measurement of neural activity, we are confident that the results of our accurate modelling framework (see methods and supplementary information) can be translated to top 1% real-life results with some confidence. Also, while modelling causal structure fitted using a pool of data collected across individuals is controversial, we only draw conclusions from single subject data aggregated through e.g. median operators. Also, while anatomical inaccuracies may confound or inference, AAL ROIs are quite “large” (compared to other atlases) and, most importantly, our final observable (median of 100 subjects) should be quite robust to such variability. In addition, of course haemodynamic convolution varies across the brain, however MVGC analysis has been seen to be robust to this confound^21^. Finally, while estimating causality in non-equilibrium time series (e.g. task-related designs) may pose some interpretability problems, all our data were resting state-data which are median-averaged before interpretation.

In conclusion, our results are in keeping with the notion that the directionality of the functional connectivity effects that typically emerge in rs-fMRI studies should be interpreted with caution unless the specific causal and directional relationship across different brain areas is explicitly assessed as we did in this study. All in all, revealing the directionality of connectivity effects in rs-fMRI studies may be not only relevant to better understand the functionality of the “healthy” brain but could also provide important hints into the pathophysiological mechanisms that lead to the development of neuro-psychiatric disorders, a vast group of illnesses in which the causal and directional connectivity patterns across several neural circuits are expected to be altered^63^.

## Methods

### Multivariate Granger Causality

Given two components *x*_*j*_(*t*) and *x*_*i*_(*t*) of time-resolved *n*-dimensional signals **x**(*t*), in order to evaluate MVGC strength *j* → *i* (hence incorporating the confounding influence of possible spurious interaction between *x*_*i*_ and *x*_*j*_ mediated by the other component of the system) - two separate VAR models are fitted. The first model (commonly termed ‘restricted model’) is an VAR model that estimates the future of (**x**(*t*)|*x*_*j*_) (the whole system excluding component *j*) as a function of its past:

1 $${({\bf{x}}(t)|j)}_{t}-{\bf{A}}{L}^{P}{({\bf{x}}(t)|j)}_{t}={\boldsymbol{\varepsilon }}\quad \quad \quad (\text{restricted}\,\text{model})$$

where *L*^*P*^ is the lag-operator, i.e. a vector of length *p* containing the *p* previous values of **x**(*t*), **A** is a (*n*−1) × *P* matrix of coefficients, and **ε** is a white uncorrelated noise process with variance **D**. The second model (commonly termed ‘unrestricted model’) is a VAR process that models all components of **x** (including *x*_*j*_) as a function of their pasts:

2 $${\bf{x}}(t)-{\bf{A}}{\boldsymbol{^{\prime} }}{L}^{P}({\bf{x}}(t))={\boldsymbol{\varepsilon }}{\boldsymbol{^{\prime} }}\quad \quad \quad \,\quad (\text{unrestricted}\,\text{model})$$

In this case **A**′ is a *n* × *P* matrix of coefficients, and **ε**′ is a white time-uncorrelated noise vector process with covariance matrix **D**′. The unrestricted model also includes the component (*x*_*j*_) in the variables which may contribute to the prediction of the future of *x*_*i*_. In this context, *j* → *i* MVGC strength can be estimated as:

3 $$\mathrm{log}\,[({{\bf{D}}}_{i,i})/({{\bf{D}}{\boldsymbol{^{\prime} }}}_{i,i})]$$

Equation (3) estimates the information gain provided by *x*_*j*_ about the future of *x*_*i*_ while excluding the information added by all other variables in the network. It is also important to note that while the definition in Equation (3) employs the diagonal elements of **D**, these are estimated while accounting for possible covariance between *x*_*i*_ and *x*_*j*_.

### Synthetic network generation

**Figure 6:**
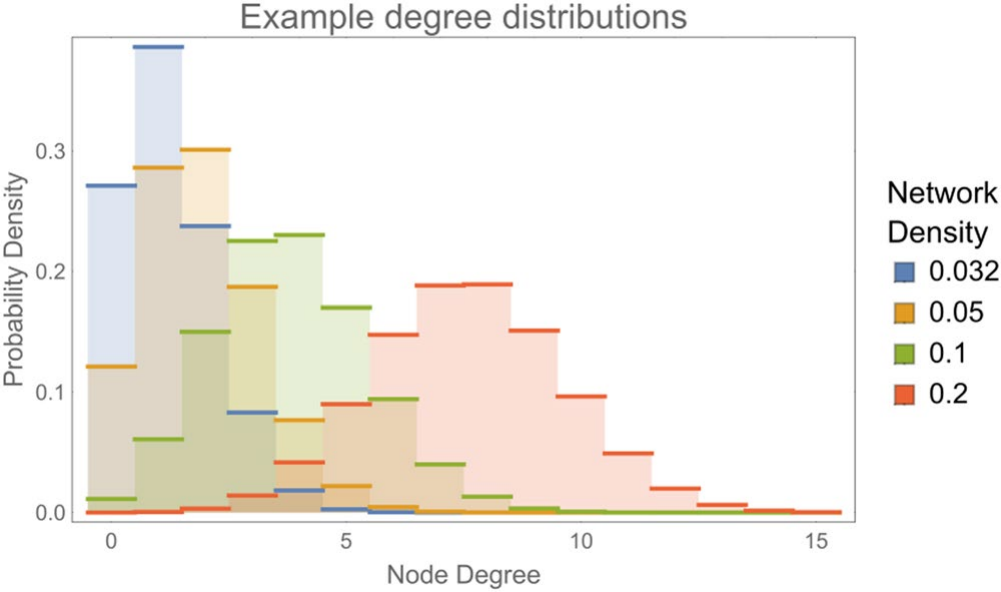
Example degree distributions for synthetic data validation. All distributions were generated for three uniform graph distributions with network densities 0.032, 0.05, 0.1 and 0.2 (from left to right, respectively).

**Figure 7:**
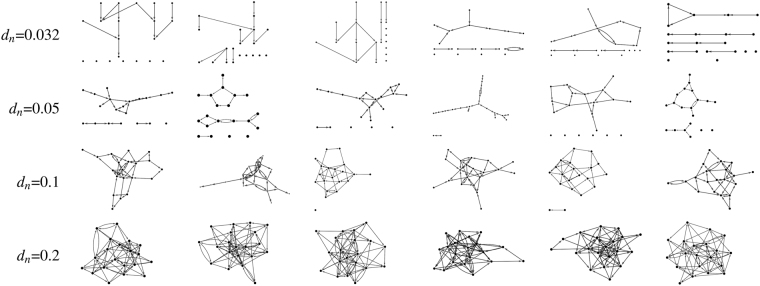
Example networks employed for synthetic simulations. On each row all networks have the same density, d_n_ = 0.032, 0.05, 0.1, 0.2, corresponding to average node degrees 1.2, 1.9, 3.8, 7.6, respectively.

In order to compare the performance of MVGC (Equation (3)) in detecting true causal connections within complex directed networks, we performed large synthetic data simulation by generating realistic BOLD data from a family of 20-node networks (see Figs 6 and 7 below).

We generated random networks from a uniform graph distribution, a procedure in which the graph is built recursively. Starting with N disconnected nodes “edges” (i.e. connections) with random direction between two not already connected nodes are randomly assigned up to the required density. It should be noted that nodes with bidirectional connections as well as loops are explicitly allowed. The total number of edges ne depends on the desired network density *d*_*n*_ which, for a network with *N* nodes, is defined as the ratio between the number of edges over total number of possible edges (the latter is *N*x(*N*−1)). After the graph has been generated, the resulting number of edges incident to each node represents the degree of that node. In this paper, we generated graphs with 23 different densities, hence corresponding to 23 total numbers of edges *n*_*e*_ = {2, 3, 4, 5, 6, 8, 10, 12, 15, 19, 24, 30, 38, 48, 60, 76, 95, 120, 151, 190, 240, 302, 380}. *n*_*e*_ values were chosen so that the corresponding densities *d*_*n*_ were approximately equidistant on a logarithmic scale between 0.005 and 1. For each network density *d*_*n*_ we randomly generated 32 different networks. Examples of the generated networks for four distinct values of network densities are shown in Fig. 7.

### Synthetic BOLD timeseries

**Figure 8:**
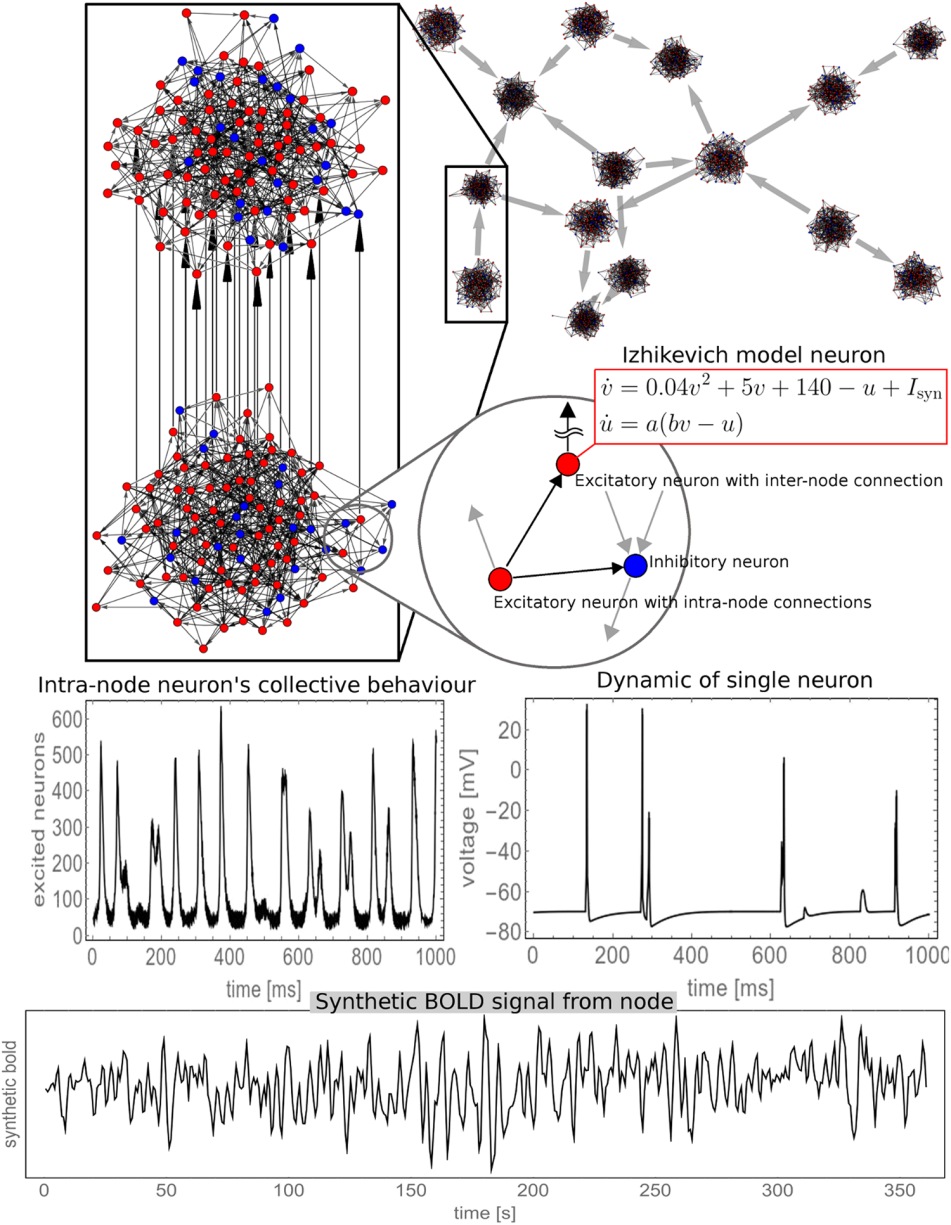
Generating synthetic BOLD signals for each node–both inter-node and intra-node coupling are depicted (see supplementary information for details).

Each node represents a small cortical volume (see Fig. 8), equipped with its own simulated neuronal dynamics where single neurons are coupled intra-node (local coupling). Nodes are also connected through simulated synaptic dynamics (inter-node coupling). The readout variable of every node is a synthetic BOLD signal derived from a physiological inspired model, which is obtained from a dynamical interplay of blood volume, blood flow and oxygen consumption^53^, dependent on neural activities, which in turn are simulated with the Izhikevich neuronal model^64,65^. Details about the synthetically generated dynamics, including the precise model and parameters, are presented and discussed in the supplementary information, and graphical summary is show on Fig. 8.

For each realization we then evaluated the MVGC strengths for both directions of every pair of nodes. The ability of detecting causal links (i.e. belonging to the ground truth network) while rejecting false causal links was quantified as the area under the receiver operating characteristic (ROC) curve (AUC). The AUC was computed by varying the threshold on the causality strength which determined whether a causal connection should be accepted as “true” or rejected. Also, when fitting VAR models the choice of the optimal AR order *P* is commonly made according to a statistical criterion, e.g. the Bayesian Information Criterion (BIC) or the Akaike Information Criterion (AIC)^66^. This is in line with the aim of obtaining the best compromise between model parsimony and goodness of fit. However, since in the context of our paper the use of the AR models serves the purpose of detecting ‘true’ top 1% causal links (i.e. having a high PPV at the top 1% connections) while retaining some specificity causality, for each density and each network we employed the order *P* (chosen between 1 and 15) which resulted in the highest average between PPV and AUC when this figure was averaged over all realization of each network. In other words, given our specific aim of detecting true causal links with high PPV only at the top 1% (which in *in vivo* data already provides a wealth of information), the sum of AUC and PPV was employed as a figure of merit for model order selection and yielded an order of 3, which was therefore also applied to human data (see below).

### Directed Connectome of the Human Brain

After synthetic validation of the suitability of MVGC we explored the structure of MVGC-based networks in the human brain by making use of 100 unrelated subjects made available by the Human Connectome Project^67^. The subjects included in this dataset underwent multi-band accelerated (repetition time TR = 0.72s) resting-state fMRI scans each, on a 3 Tesla scanner with an isotropic spatial resolution of 2 mm. Each subject underwent four scanning sessions consisting of 1200 volumes each^39^. Preprocessing details, which include corrections for spatial distortions and head motion, registration to the T1-w structural image, resampling to 2 mm MNI space, global intensity normalization, high-pass filtering and FIX artefact removal procedure can be found in^68^. After pre-processing the average BOLD signal was extracted in 116 regions of interest (ROIs) using the Automated Anatomical Labelling atlas^69^. Prior to causality analysis, we tested for stationarity of timeseries, i.e. we tested the hypothesis that the probability density function (PDF) of the signal distribution is not function of time. In detail, for each subject *k*, for each ROI, have partitioned the timeseries into 3-minute windows (chosen as a compromise between data quantity and series shortness) and used the Kolmogorov-Smirnov test to check whether the PDF of any of these segments was statistically different (p < 0.05) than the PDF of the whole signal it was extracted from. Over all subjects and all brain regions, approximately 7% of all the 3-minutes windows resulted to be distributed in a statistically significantly different way from the full timeseries, which for practical terms covered against stationarity issues. For each subject *k*, for each pair of ROIs and for each direction we evaluated MVGC strength (see Equation (3)), obtaining a 116 × 116 non-symmetric matrix of MVGC strength ***G***_*k*_ whose diagonal elements are set to zero. Accordingly to results from our realistic simulation of the BOLD signal, the autoregressive model order for MVGC estimations was set to 3. It is important to note that the total number of parameters (when estimating the model and covariance Matrix using the Burg algorithm) is (for the unrestricted model) = 116 × 116 × 3 + 116 × (116 + 1)/2 = 47154. In turn, the available datapoints for each subjects are 116 × 1200 × 4 = 556800, which results in approximately 12 datapoints per parameter.

Subject-specific matrices ***G***_*k*_ were then aggregated into the intersubject median matrix ***G****.* Successively we retained only the connections belonging to ***G*** corresponding to the top 1% in MVGC strength. This thresholding operation was performed in order to ensure a low false positive rate and high true positive rate in absence of knowledge about the ground truth (i.e. without prior knowledge of the “true” causal connectome of the brain). Additionally, in order to explicitly explore the direction of causality within ***G***, we defined the normalized causality “flow” ***F*** as the antisymmetrical part of ***G*** rescaled by the symmetrical part of ***G***, i.e. ***F***_*i,j*_ = (***G*** − ***G***^T^)_*i,j*_/(***G* + *G***^T^)_*i,j*_. The normalized causality “flow” ***F*** summarizes the prevailing direction of causality, net of changes in overall causality strength.

## Electronic supplementary material

Supplementary Information
