# Supplementary material for: Multivariate Granger causality unveils directed parietal to prefrontal cortex connectivity during task-free MRI
Source: Sci Rep. 2018 Apr 3;8:5571. doi: 10.1038/s41598-018-23996-x (PMC5882904; doi:10.1038/s41598-018-23996-x)
Supplement: Supplementary file 1 — Supplementary Information [file 41598_2018_23996_MOESM1_ESM.doc]

**Multivariate Granger causality unveils directed parietal to prefrontal cortex connectivity during task-free MRI**

Andrea Duggento^1^, Luca Passamonti^2,3^, Gaetano Valenza^4^, Riccardo Barbieri^5,6^, Maria Guerrisi^1^ and Nicola Toschi^1,7^

**Authors’ institutional affiliations:**

^1^Department of Biomedicine and Prevention, University of Rome Tor Vergata, Rome, Italy

^2^Institute of Bioimaging and Molecular Physiology, National Research Council, Catanzaro, Italy

^3^Department of Clinical Neurosciences, University of Cambridge, Cambridge, UK

^4^Bioengineering and Robotics Research Centre “E. Piaggio”, and the Department of Information Engineering, University of Pisa, Pisa, Italy.

^5^Department of Electronics, Informatics and Bioengineering, Politecnico di Milano, Milano. Italy.

^6^Department of Anesthesia, Massachusetts General Hospital, Boston, Massachusetts, USA

^7^Department of Radiology, Athinoula A. Martinos Center for Biomedical Imaging, Massachusetts General Hospital and Harvard Medical School, Boston, Massachusetts, USA

**Corresponding Author:** Dr. Andrea Duggento**,** Email: duggento@med.uniroma2.it

**Supplementary information**

**Neuronal Dynamics --** Spiking dynamics of each neuron and is simulated by using the Izhikevich model, which is expressed by a fast variable *v* and a slow recovery variable *u*:

in particular, *v* is the membrane potential (in mV), while *u* approximates the difference of all inward and outward voltage-gated currents. *I_syn_* is the total synaptic current, and *a* and *b* are parameters. When the membrane potential reaches the peak of the spike, i.e. *v*=30 mV, the model variables are reset according to *v=c* and *u=d*, where *c* and *d* are model parameters. Infra-node heterogeneity between neurons is achieved by varying the parameters *a*, *b*, *c* and *d.* Details of parameters distribution also employed in this simulation can be found in[^2^](#_ENREF_2). This heterogeneous distribution, which distinguish between excitatory and inhibitory neurons, reflects the distribution of neuronal types of the majority of mammalian neocortex[^3^](#_ENREF_3).

At each node a total of 1000 neurons were simulated, while keeping a 1:4 ratio between inhibitory and excitatory neurons[^4^](#_ENREF_4).

In addition, neuronal dynamics for the *i*th neuron also encompass its synaptic conductance dynamics:

$$I_{\text{syn}}=g_{\text{AMPA}}v + g_{\text{NMDA}}\frac{[(v+80)/60]^{2}}{1+[(\mathrm{v}+80)/60]^{2}}v+g_{\text{GABA}_{\text{A}}}(v+70)+g_{\text{GABA}_{\text{B}}}(v+90)$$

were each `*g’*s constant represent the conductance of four receptors types (AMPA, NMDA, GABA_A_, GABA_B_) which follow their own dynamics: , where τ = 5, 150, 6 and 150 ms for AMPA, NMDA, GABA_A_ and GABA_B_ receptors respectively.

**Local, intra-node coupling –** each neuron (both excitatory and inhibitory) has a fixed probability *p_intra_*=0.2 of establishing a directed connection to every other neuron of the same node; a fixed probability *p_intra_* was chosen to keep the average number of intra-node connection equal for all neurons (200 connections per neuron in average) while allowing variability between neurons. If a presynaptic neuron *j* is connected with postsynaptic neuron *i*, synaptic firing of *j* produces in *i* an increase of some *g* conductances by *c_c_R_j_w_j_*, where *c_c_* is a coupling constant, *R* is a ‘depression’ variable and *w* is a ‘facilitation’ variable[^5^](#_ENREF_5). If the presynaptic neuron is an excitatory type, *g*_AMPA_ and *g*_AMDA_ are updated, while if the presynaptic neuron is an inhibitory type, *g*_GABAA_ and *g*_GABAB_ are updated. ‘Depression’ variable R and ‘facilitation’ variable *w* that define synaptic plasticity also have their own dynamics (see model by Markram et al.[^5^](#_ENREF_5)):

*U*, *F* and *D* are parameters experimentally measured in literature. *U*, *F* and *D* are specific for each neuron types (excitatory- or inhibitory-type neurons).

**Inter-node coupling**

The ground truth adjacency matrix *A* is used to directly connect two nodes. "Presynaptic" and "postsynaptic" are, respectively, the driving (causal source) and the driven (causal sink) nodes. From a presynaptic node, *n_out_*=15 excitatory neurons are chosen at random and a directed (inter-node) connection is established between them and each of the neuron (excitatory and inhibitory) in the post-synaptic node with probability *p_inter_*=*p_intra_*=0.2. *n_out_* was chosen such that the number of outward connect neurons was low with respect to the total excitatory neurons in the same node (even in the case of a node connected towards every other node). The inter-node connection is implemented identical to the local coupling, except that the effects on the postsynaptic neuron are delayed by 10 ms from the onset of the spike on the presynaptic neuron. To stress the symmetry between intra- and inter-node coupling the probabilities *p_inter_*=*p_intra_* were arbitrary set to the same value. If a presynaptic node is connected to more than one postsynaptic node, for each extra inter-node connection the presynaptic neurons are chosen among those not previously engaged in inter-node connection towards other nodes, i.e. each single presynaptic neuron can only be connected to postsynaptic neurons of one node.

**BOLD dynamics**

We describe BOLD signal as described in [^7^](#_ENREF_7). The BOLD signal *y* is a function of normalized venous volume *V*, normalized total deoxyhemoglobin *q,* and the basal rate of oxygen extraction *E­_0_*, and resting blood volume fraction *V_0_*:

$$\begin{matrix} y(t)=V_{0}(k_{1}(1-q)+k_{2}(1-q/V)+k_{3}(1-V)) \\ \mathrm{k}_{1}=7E_{0} \\ \mathrm{k}_{2}=2 \\ \mathrm{k}_{3}=2E_{0}-0.2 \end{matrix}$$

While *q* and *V* are time dependent variables with their own dynamics, *V_0_* and *E_0_* are assumed to be constant. The rate of normalized volume follow:

Here, the rate of change of volume *V* is proportional to the difference of the inflow *f_in_* and the outflow *f_out_* ,which in turn depends on volume *V.* Also, the rate of change of deoxyhemoglobin *q* is proportional to the difference between the rate of import minus the rate of extraction:

Where *E/E_0_* represents the ratio of extracted oxygen, which is a function of the flow and the oxygen extraction[^8^](#_ENREF_8):

$$E=1-(1-E_{0})^{\frac{1}{f_{in}}}$$

**Neurovascular coupling**

The system depends on the inflow *f_in_,* whose dynamics is to be specified. Following[^9^](#_ENREF_9) we assume that a local neurovascular signal *s* is responsible for the rate of change of *f_in_*:

$$\mathop{f_{in}}\limits^{.} =s$$

where *s* has a specified dynamics:

The first term of the r.h.s. specify that the rate of change of *s* is proportional to the neuron activity *u*, the second and the third terms are relaxation terms with respect to *s* itself and the inflow *f_in_*. We now only need to define the neuron activity *u* in terms of variables which describe the Izhikevich-neuron; since the BOLD signal is known to depend on the conductances of AMBA receptor types (which contribute to local field potential modulation) and of NMDA receptor types (which modulates blood flow through nitric oxide signaling pathway), we set the neuron activity *u* as:

$u=\sum_{j} g_{AMPA}+g_{NMDA}$.

*u* is therefore the node "signal" which enters causality estimation.

**Numerical values for constant parameters**

Table of variables used in the simulation:

| name | value | unit |
| --- | --- | --- |
| *U_exc_* | 0.5 | ms^-1^ |
| *F_exc_* | 1000 | ms |
| *D_exc_* | 800 | ms |
| *U_inh_* | 0.2 | ms^-1^ |
| *F_inh_* | 20 | ms |
| *D_inh_* | 700 | ms |
| *V_0_* | 0.02 | a.u. |
| *E_0_* | 0.8 | a.u. |
| α | 0.2 | a.u. |
| τ_s_ | 800 | ms |
| τ_0_ | 1000 | ms |
| τ_f_ | 400 | ms |
| ϵ | 0.0001 | a.u. |

**Bibliography**

1 Izhikevich, E. M. Simple model of spiking neurons. *Ieee T Neural Networ* **14**, 1569-1572, doi:10.1109/Tnn.2003.820440 (2003).

2 Izhikevich, E. M. Which model to use for cortical spiking neurons? *Ieee T Neural Networ* **15**, 1063-1070, doi:10.1109/Tnn.2004.832719 (2004).

3 Steriade, M., Timofeev, I. & Grenier, F. Natural waking and sleep states: A view from inside neocortical neurons. *J Neurophysiol* **85**, 1969-1985 (2001).

4 Izhikevich, E. M., Gally, J. A. & Edelman, G. M. Spike-timing dynamics of neuronal groups. *Cerebral cortex* **14**, 933-944, doi:10.1093/cercor/bhh053 (2004).

5 Markram, H., Wang, Y. & Tsodyks, M. Differential signaling via the same axon of neocortical pyramidal neurons. *Proceedings of the National Academy of Sciences of the United States of America* **95**, 5323-5328, doi:DOI 10.1073/pnas.95.9.5323 (1998).

6 Gupta, A., Wang, Y. & Markram, H. Organizing principles for a diversity of GABAergic interneurons and synapses in the neocortex. *Science* **287**, 273-278, doi:DOI 10.1126/science.287.5451.273 (2000).

7 Buxton, R. B., Wong, E. C. & Frank, L. R. Dynamics of blood flow and oxygenation changes during brain activation: The balloon model. *Magnetic resonance in medicine* **39**, 855-864, doi:DOI 10.1002/mrm.1910390602 (1998).

8 Buxton, R. B., Uludag, K., Dubowitz, D. J. & Liu, T. T. Modeling the hemodynamic response to brain activation. *NeuroImage* **23 Suppl 1**, S220-233, doi:10.1016/j.neuroimage.2004.07.013 (2004).

9 Friston, K. J., Mechelli, A., Turner, R. & Price, C. J. Nonlinear responses in fMRI: the Balloon model, Volterra kernels, and other hemodynamics. *NeuroImage* **12**, 466-477 (2000).

10 Brette, R. & Destexhe, A. *Handbook of neural activity measurement*. (Cambridge University Press, 2012).

11 Logothetis, N. K. What we can do and what we cannot do with fMRI. *Nature* **453**, 869 (2008).

12 Akgören, N., Fabricius, M. & Lauritzen, M. Importance of nitric oxide for local increases of blood flow in rat cerebellar cortex during electrical stimulation. *Proceedings of the National Academy of Sciences* **91**, 5903-5907 (1994).

13 Li, J. & Iadecola, C. Nitric oxide and adenosine mediate vasodilation during functional activation in cerebellar cortex. *Neuropharmacology* **33**, 1453-1461 (1994).
